# Emergence of a Novel Recombinant Norovirus GII.P16-GII.12 Strain Causing Gastroenteritis, Alberta, Canada

**DOI:** 10.3201/eid2508.190059

**Published:** 2019-08

**Authors:** Kanti Pabbaraju, Anita A. Wong, Graham A. Tipples, Xiaoli-Li. Pang

**Affiliations:** Public Health Laboratory (ProvLab),; Alberta Public Laboratories, Edmonton, Alberta, Canada (K. Pabbaraju, A.A. Wong, G.A. Tipples, X.-L. Pang);; University of Alberta, Edmonton, Alberta, Canada (G.A. Tipples, X.-L. Pang)

**Keywords:** norovirus, GII.P16-GII.12, Alberta, Canada, recombinant, acute gastroenteritis, viruses, enteric infections

## Abstract

We identified a novel recombinant GII.P16-GII.12 norovirus associated with epidemic and endemic gastroenteritis during March 1, 2018–February 12, 2019, in Alberta, Canada. GII.12 viruses have not been detected in Alberta since 2000. Comparing the full genome of this strain to previously published sequences revealed this virus to be a novel recombinant strain.

Norovirus is the leading cause of epidemic and endemic acute gastroenteritis (AGE) worldwide. Norovirus can evade the host immune response by accumulating mutations that have a biological advantage by antigenic drift (*1*). In addition, recombination at the junction of open reading frame (ORF) 1 and 2 can result in the circulation of a novel strain. 

In the past 2 decades, emerging genetic clusters of norovirus GII.4 were associated with epidemics in Alberta, Canada. Multiple GII.4 viruses were associated with epidemics during the 2000s. Since 2010, a single variant, GII.4 Sydney, has been the predominant virus (*2*–*5*). A recombinant GII.P16-GII.4 Sydney strain emerged in July 2015 and caused 72% of the outbreaks in the winter of 2017–18 (*2*). A small wave of activity with the Kawasaki GII.17 and GII.P16-GII.2 strains was seen during 2016–2017, but they did not predominate.

The Alberta Molecular Surveillance Program includes genotyping of 1 norovirus-positive stool sample from each outbreak for the early detection of novel strains. During March 1, 2018–February 12, 2019, we report the detection of a novel recombinant GII.P16-GII.12 that was identified in May 2018 and caused AGE outbreaks and sporadic cases in children <6 years of age.

## The Study

Public health officials and the Public Health Laboratory (ProvLab) in the province of Alberta use established protocols to investigate all suspected AGE outbreaks. Stool samples are tested for norovirus genogroups I and II using a real-time reverse transcription PCR (*4*). An outbreak includes >2 epidemiologically linked AGE cases with >1 norovirus-positive sample. ProvLab genotyped 1 norovirus-positive sample from each outbreak and those from children with sporadic AGE by using the dual polymerase-capsid genotyping protocol (*6*). In our study, 72/108 (67%) AGE outbreaks had test results positive for norovirus, of which 5 (7%) were identified as norovirus GI, 66 (92%) as GII, and 1 (1%) as GI and GII (Table 1). During the same period, 94/755 (12%) AGE cases in children had test results positive for norovirus, 6 as GI and 88 as GII. 

**Table 1:** Investigation of outbreaks of acute gastroenteritis, March 1, 2018–February 12, 2019, Alberta, Canada

Month	Outbreaks	Norovirus-positive, no. (%)	GI	GII	GI and GII
Mar	16	11 (69)	1	10	0
Apr	9	8 (89)	1	7	0
May	4	2 (50)	1	1	0
Jun	2	0 (0)	0	0	0
Jul	3	1 (33)	0	1	0
Aug	3	1 (33)	0	0	1
Sep	3	1 (33)	0	1	0
Oct	4	3 (75)	0	3	0
Nov	12	8 (67)	0	8	0
Dec	26	17 (65)	0	17	0
Jan	21	15 (71)	2	13	0
Feb	5	5 (100)	0	5	0
Total	108	72 (67)	5	66	1

**Figure 1:**
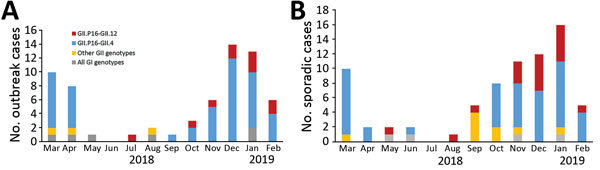
Monthly trends of norovirus genotypes for outbreak (A) and sporadic (B) cases of acute gastroenteritis in Alberta, Canada, during March 2018–February 2019. Genotypes included under other are listed in Table 2.

We genotyped 64/72 (89%) outbreaks and 74/94 (79%) sporadic cases and detected diverse genotypes and different monthly trends (Table 2; Figure 1). GII.P16-GII.4 Sydney 2012 was the predominant strain followed by GII.P16-GII.12 in both outbreaks and sporadic AGE, but distribution of other genotypes was different, most notably GII.P16-GII.2 and GII.P12-GII.3 strains were detected in the sporadic cases but not in outbreaks (Table 2). 

**Table 2:** Genotyping results for outbreak and sporadic cases of acute gastroenteritis, March 1, 2018–February 12, 2019, Alberta, Canada

Characteristics	Outbreak	Sporadic	Total
No. cases	72	94	166
No. (%) genotyped	64 (89)	74 (79)	138 (83)
GI genotypes, no. cases			
GI.P2-GI.2	0	1	1
GI.P3-GI.3	3	2	5
GI.Pb-GI.6	1	0	1
GI.P6-GI.6*	1	0	1
GI.P7-GI.7	0	1	1
GI.PUnknown-GI.7	1	0	1
GII genotypes, no. cases			
GII.P4-GII.4	1	1	2
GII.P7-GII.6	1	0	1
GII.P7-GII.14*	1	0	1
GII.P12-GII.3	0	4	4
GII.P16-GII.2	0	2	2
GII.P16-GII.4	46	44	90
GII.16-GII.12	10	17	27
GII.P-unknown_GII.12	0	2	2

*Co-infection with genogroups GI and GII.

The GII.P16-GII.2 strain emerged in 2016 and caused a large norovirus epidemic in Asia (*7*,*8*), followed by a small wave in Alberta in winter 2016–17 (*2*), but this strain was not detected in outbreaks during our study. Of note, the emerging GII.P16-GII.12 strain became the second most predominant strain with increasing cases, especially in sporadic AGE, after November 2018 (Figure 1, panel B). This strain caused 80% (8/10) of outbreaks in long-term care facilities and 2 in hospital acute-care units. Of the 17 sporadic cases associated with this strain, we retrieved patient information for 14 cases; 12 were hospitalized, 1 was seen in the emergency department, and 1 was an outpatient. The number of hospitalized patients probably indicates more severe disease associated with this strain. However, because the number of cases was small, further study and caution in interpretation of disease severity is warranted.

We sequenced the near-complete genome of the novel GII.P16-GII.12 strain from 2 different outbreaks by Sanger sequencing using primers designed in-house (available upon request). We performed contig assembly with Seqscape v2.7 (Advanced Biosystems, https://seqscape.software.informer.com/2.7) and sequence alignments with ClustalW (http://www.clustal.org/clustal2). For phylogenetic analysis, we inferred evolutionary history by using the neighbor-joining method. We computed evolutionary distance by using the maximum composite likelihood model for nucleotide sequences and the Poisson model for amino acid sequences. We performed a bootstrap test by using 1,000 replicates in MEGA6 (http://www.megasoftware.net) (*9*). We obtained 7,406 bp and submitted the sequences to GenBank (accession nos. MK355712–3).

**Figure 2:**
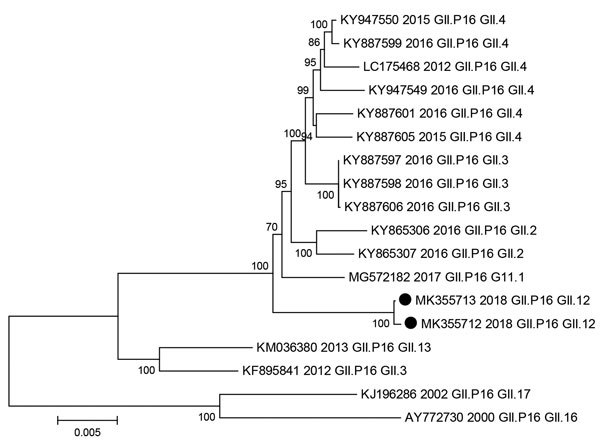
Phylogenetic tree of open reading frame 1 for norovirus GII.P16 strains. Black dots indicate nucleotide sequences of novel GII.P16-GII.12 strains identified in Alberta, Canada, during March 2018–February 2019. GenBank accession numbers and year identified are provided. Scale bar indicates nucleotide substitutions per site.

We compared the ORF1 sequence to the RIVM (http://www.rivm.nl/mpf/norovirus/typingtool) and National Center for Biotechnology Information (http://www.ncbi.nlm.nih.gov) databases. The closest sequence match was from GII.P16-GII.4 Sydney 2012 (accession no. LC175468), the current predominant genotype worldwide (*6*,*10*). At the nucleotide level, ORF1 of MK355712 shared 96.92% identity with the Sydney 2012 strain and MK355713 shared 96.98% identity. At the amino acid level, ORF1 of MK355712 shared 98.56% identity and MK355713 shared 98.65% identity with the Sydney 2012 strain. We constructed a phylogenetic tree comparing the near full-length ORF1 of the strains we report with the GII.P16 sequence associated with the viral protein (VP) 1 capsid region from different genotypes (Figure 2). The ORF1 clustered with the ORF1 from contemporary strains including GII.4 Sydney, GII.2, and the recently described GII.3 and GII.1 sequences (accession nos. KY887597 and MG572182), but our sequences lie on an independent branch.

GII.P16 sequences from this study clustered with GII.P16 sequences that form a previously reported distinct monophyletic clade with a common ancestor from 2013 (*10*) but lie on an independent branch (data not shown). Others have described the GII.P16 sequences belonging to this clade as containing nonsynonymous substitutions in ORF1 along the branch leading to the common ancestor of the GII.P16-GII.4 Sydney 2012/GII.3 clade, several of which occur close to positions known to affect polymerase function and transmission (*10*). We saw amino acid changes in our sequences that were also noted in the GII.Pe and GII.P16 polymerase of GII.4 Sydney viruses, including D173E, G163A, L337M, and S502N, but we did not see the K1646R unique to GII.P16 associated with GII.4 Sydney. We noted some of the point mutations in our sequences that have been described on the polymerase surface of novel GII.P16 strains, including K357Q, T360A, and V332I (*6*,*7*,*10*,*11*), which caused re-emergence of a new cluster. Because we did not see all the known mutations, effects of these amino acid changes will require further investigation.

We compared ORF2 and ORF3 to the GenBank database using BLAST (https://blast.ncbi.nlm.nih.gov/Blast.cgi) and found the closest sequence matches are contemporary strains described in Taiwan (*12*), Australia (*13*), and the United States (*14*,*15*). Identity with these sequences were 93%–94% at the nucleotide level and 98%–99% at the amino acid level in ORF2 and 84%–85% at the nucleotide level and 86%–87% at the amino acid level in ORF3. Identity with the prototype Wortley strain (accession no. AJ277618) was lower. Our sequences demonstrated amino acid changes reported in the contemporary GII.12 strains, including A22V, I47V, and S465T, but we did not see the N392S described in all sequences of the GII.12 strain cluster in our sequence (*15*). The P2 region of VP1 has the antigenic and histoblood group antigen attachment sites, and 3 unique amino acid substitutions, including N350D, A352T, and I361V were seen in this region. Studies to explore whether these mutations play a role in binding profiles and immunity will help us understand if this virus is under selective pressure, which can give rise to novel variants.

## Conclusions

We describe a novel recombinant GII.P16-GII.12 norovirus strain identified in Alberta, Canada. Although the GII.P16-GII.4 strain is still predominant, this novel strain seems to be playing a role in both outbreaks and sporadic cases in young children. Continued surveillance and prompt genotyping are critical to monitor the emergence and prevalence of novel norovirus strains.
